# Treatment potential of LPCN 1144 on liver health and metabolic regulation in a non-genomic, high fat diet induced NASH rabbit model

**DOI:** 10.1007/s40618-021-01522-7

**Published:** 2021-02-13

**Authors:** P. Comeglio, E. Sarchielli, S. Filippi, I. Cellai, G. Guarnieri, A. Morelli, G. Rastrelli, E. Maseroli, S. Cipriani, T. Mello, A. Galli, B. J. Bruno, K. Kim, K. Vangara, K. Papangkorn, N. Chidambaram, M. V. Patel, M. Maggi, L. Vignozzi

**Affiliations:** 1 Andrology, Women’s Endocrinology and Gender Incongruence Unit, Department of Experimental and Clinical Biomedical Sciences “Mario Serio”, University of Florence, Viale Pieraccini, 6, 50139 Florence, Italy; 2 Section of Human Anatomy and Histology, Department of Experimental and Clinical Medicine, University of Florence, Florence, Italy; 3 Interdepartmental Laboratory of Functional and Cellular Pharmacology of Reproduction, Department of Neurosciences, Psychology, Drug Research and Child Health (NEUROFARBA), University of Florence, Florence, Italy; 4 Gastroenterology Unit, Department of Experimental Clinical and Biomedical Sciences “Mario Serio”, University of Florence, Florence, Italy; 5 Lipocine Inc., Salt Lake City, Utah 84088 USA; 6 Endocrinology Unit, Department of Experimental Clinical and Biomedical Sciences “Mario Serio”, University of Florence, Florence, Italy; 7 I.N.B.B. (Istituto Nazionale Biostrutture E Biosistemi), Rome, Italy

**Keywords:** Testosterone, Liver, NASH, Inflammation, Steatosis, Fibrosis

## Abstract

**Purpose:**

Low free testosterone (T) level in men is independently associated with presence and severity of Non-Alcoholic Steatohepatitis (NASH). The histological and molecular effects of oral testosterone prodrug LPCN 1144 treatment on hepatic fibrosis and NASH features are unknown. A metabolic syndrome-induced NASH model in rabbits consuming high fat diet (HFD) has been previously used to assess treatment effects of injectable T on hepatic fibrosis and NASH features. Here we present results on LPCN 1144 in this HFD-induced, NASH preclinical model.

**Methods:**

Male rabbits were randomly assigned to five groups: regular diet (RD), HFD, HFD + 1144 vehicle (HFD + Veh), HFD + 1144 (1144), and HFD + 1144 + α-tocopherol (1144 + ALPHA). Rabbits were sacrificed after 12 weeks for liver histological, biochemical and genetic analyses. Histological scores were obtained through Giemsa (inflammation), Masson’s trichrome (steatosis and ballooning), and Picrosirius Red (fibrosis) staining.

**Results:**

Compared to RD, HFD and HFD + Veh significantly worsened NASH features and hepatic fibrosis. Considering HFD and HFD + Veh arms, histological and biomarker features were not significantly different. Both 1144 and 1144 + ALPHA arms improved mean histological scores of NASH as compared to HFD arm. Importantly, percentage of fibrosis was improved in both 1144 (*p* < 0.05) and 1144 + ALPHA (*p* = 0.05) treatment arms vs. HFD. Both treatment arms also reduced HFD-induced inflammation and fibrosis mRNA markers. Furthermore, 1144 treatments significantly improved HFD-induced metabolic dysfunctions.

**Conclusions:**

Histological and biomarker analyses demonstrate that LPCN 1144 improved HFD-induced hepatic fibrosis and NASH biochemical, biomolecular and histochemical features. These preclinical findings support a therapeutic potential of LPCN 1144 in the treatment of NASH and of hepatic fibrosis.

## Introduction

Metabolic syndrome (MetS) is a cluster of metabolic abnormalities, including hypertension, insulin resistance, impaired glucose tolerance and visceral obesity [1]. In MetS, adipose tissue mass is increased, and adipocytes have a reduced metabolic capacity to store surplus energy, thus becoming severely dysfunctional and insulin resistant [2]. In fact, the impaired differentiation of preadipocytes, driven by insulin resistance, results in enlarged mature adipocytes that are unable to store excess lipids [3]. Insulin resistance in adipocytes is the prominent force behind MetS development and is considered a pivotal feature in distinguishing between a ‘metabolically healthy’ from a ‘metabolically unhealthy’ obesity [4, 5]. Moreover, MetS is accompanied by dyslipidemia, with elevated triglyceride and cholesterol levels [6], being the dysfunctional adipose tissue the major contributor to the increased triglyceride accumulation [7]. The excess of circulating triglycerides ultimately leads to fat deposition and inflammation within other tissues involved in metabolic homeostasis, such as liver, wherein ectopic fat deposition amplifies insulin resistance and interferes with several cellular functions [8, 9]. Intrahepatic triglyceride overload, along with the activation of inflammatory and fibrogenic pathways, characterizes a spectrum of hepatic manifestations covered under the umbrella term of non-alcoholic fatty liver disease (NAFLD). Insulin resistance has been implicated in both the initiation of NAFLD and of its progression towards NASH, and it is seen as the underlying mechanism linking visceral adipose tissue dysfunction to NASH in MetS [10–12]. NAFLD is therefore considered the hepatic hallmark of the insulin resistance associated with MetS [13–16]. Epidemiological and pre-clinical studies indicate that NASH patients are at higher risk for cardiovascular disorders, independent of underlying cardiometabolic risk factors [17–20]. Therefore, NAFLD is not simply a disorder linked to MetS, but may also exacerbate MetS-associated cardiovascular events, possibly via releasing proatherogenic inflammatory molecules [21].

Our lab developed a non-genomic, high fat diet (HFD)-induced, rabbit animal model of MetS that closely resembles the human MetS phenotype, including the onset of hypogonadotropic hypogonadism [2, 22–25]. Feeding rabbits a HFD for twelve weeks induced all the classic components of MetS, as well as severe histological alterations within the liver associated with NASH, i.e. severe inflammation, lipid accumulation and fibrosis [24, 25]. HFD-induced MetS animals with NASH (also considered as Metabolic Associated Fatty Liver Disease: MAFLD) [26] were insulin resistant, as demonstrated by an impaired glucose tolerance, as compared to rabbits fed a regular diet (RD) [2, 24, 25]. A visible collagen deposition, forming pro-fibrotic septa, was evident at sites where fatty degeneration of hepatocytes occurred [24, 25]. Furthermore, HFD induced in liver homogenates a significant increase in the mRNA expression of several pro-inflammatory and pro-fibrotic markers [24, 25].

The visceral adipose tissue (VAT) isolated from these animals is characterized by insulin-resistant preadipocytes with impaired lipid handling, mitochondrial function and adipogenesis [27] as well as several alterations of the skeletal muscle, as demonstrated by histochemical and molecular analysis of the quadriceps femoris muscle from RD and HFD rabbits [28]. Furthermore, HFD-induced metabolic derangements and hypothalamic inflammation were associated with an impairment in the neurotransmitter network controlling GnRH, thus elucidating the pathogenic link between MetS and hypogonadotropic hypogonadism [29].

In this rabbit model of NAFLD, we previously showed that injectable testosterone enanthate administration (30 mg/kg, weekly for 12 weeks) favors a more healthy metabolic profile, accompanied by a significant reduction of visceral fat accumulation and of insulin resistance [27]. Moreover, this treatment normalized the HFD-induced NASH and improved liver inflammation, also reducing TNFα mRNA expression and circulating TNFα [24], a key cytokine involved in the progression from NAFLD to NASH.

The aim of this study was to evaluate the preventive effects of new oral androgen (LPCN 1144) on the development of metabolic, histomorphological, biochemical and molecular abnormalities observed in the aforementioned HFD-induced NASH in the MetS rabbit model. LPCN 1144 is an esterized oral testosterone prodrug with aliphatic fatty acid chain (testosterone undecanoate) absorbed via lymphatic route to avoid the first-pass liver metabolism. In addition, to evaluate the potential additive/synergic effects, a subset of animals was treated with a combination of LPCN 1144 and α-Tocopherol, a lipophilic vitamin considered as a potential treatment for NASH [30]. Beyond analysis of plasma markers of MetS and NASH, we included molecular (mRNA) and histological analyses.

## Methods

### Experimental plan

Male New Zealand White rabbits (Charles River, Calco, Lecco, Italy), weighing about 3 kg, were individually caged under standard conditions in a temperature and humidity-controlled room on a 12-h light/dark cycle. Water and food were unrestricted throughout the study. New Zealand White rabbits are recognized as the experimental model primary choice, since they consistently show exhaustive HFD-induced MetS hallmarks after 12 weeks of HFD [31].

After 1 week of standard diet, animals were randomly assigned to the following groups:

- RD: Control rabbits continued to receive a regular diet for 12 weeks (*n* = 10);
- HFD: Rabbits received a high fat diet (RD fortified with cholesterol and peanut oil – see below for details) for 12 weeks (*n* = 10);
- 1144: Rabbits received a HFD and were treated with oral androgen LPCN 1144 (100 mg/kg/day by oral gavage) for 12 weeks (*n* = 8);
- 1144 + ALPHA: Rabbits received a HFD and were treated with a combination of LPCN 1144 and α-Tocopherol (100 mg/kg/day and 131 mg/kg/day, respectively, by oral gavage) for 12 weeks (*n* = 8); and
- HFD + Veh: Rabbits received a HFD and were treated with the LPCN 1144 vehicle formulation only (same volume by oral gavage) for 12 weeks (*n* = 8).

The diet specifications are reported in Table 1.

**Table 1:** Experimental model diet specifications

Composition	Regular diet (RD)	High fat diet (HFD)
Water (%)	12.0	12.0
Protein (%)	16.5	12.6
Vegetable-derived fat (%)	3.5	6.0
Animal-derived fat (%)	0.0	0.5
Fiber (%)	15.5	21.2
Ash (%)	8.5	9.2

The LPCN 1144 dose employed was established based on pharmacokinetics studies in rats performed by Lipocine, demonstrating that a 1144 dose of 200 mg/kg/day, equivalent to 100 mg/kg/day in rabbits [32], determines circulating testosterone (T) levels comparable to human physiological concentrations. In addition, the plasma T levels obtained using 100 mg/kg/day of 1144 result comparable with the levels obtained with injectable T administration (30 mg/kg/week) in our previous studies [23, 27, 28, 33].

At the end of study, the rabbits were sacrificed by a lethal dose of sodium thiopental (200 mg/kg i.v.), and livers were harvested and appropriately stored at − 80 °C for the subsequent analyses. Visceral fat, prostate, seminal vesicles, skeletal muscle, kidney, heart and lung specimens were also collected and conserved at − 80 °C for further analyses if appropriate.

One animal belonging to the HFD + Veh group died prematurely (at 7 weeks and 2 days), presumably due to the excessive lipid content in the blood.

### MetS evaluation

The oral glucose tolerance test (OGTT) was performed in accordance with the published method [22]. Briefly, after an overnight fast, a 50% glucose solution was orally administered to the animals at a dose of 1.5 g/kg. Blood samples were collected via the marginal ear vein before and 15, 30, and 120 min after glucose loading. The incremental area under the curve (AUC) was calculated using the GraphPad Prism software version 5.0 for Windows (GraphPad Software, La Jolla, CA, USA).

Blood samples for glucose, total cholesterol, triglycerides, insulin-like growth factor-1 (IGF-1), bilirubin, albumin, sex-hormone binding globulin (SHBG), testosterone, and liver enzymes (ALP, GGT, AST and ALT) analyses were obtained from the marginal ear vein at week 12, in all groups. All blood samples were collected in standard conditions, before 10:00 AM after an overnight fasting. The blood was immediately centrifuged at 1800×*g* for 20 min, and collected plasma/serum stored at − 80 °C until assayed.

Plasma T levels were measured by ECLIA (ElectroChemiLuminescence ImmunoAssay) using the Elecsys Testosterone II Kit with an automated chemiluminescence system (Cobas 800; both Roche Diagnostics GmbH, Mannheim, Germany), after appropriate extraction.

Mean arterial blood pressure (MAP) was measured using a polyethylene catheter inserted into a femoral artery at week 12, after ketamine (10 mg/kg i.v.) and sodium thiopental (50 mg/kg i.v.) sedation.

Triglycerides liver content was evaluated using the Triglyceride Quantification Colorimetric/Fluorometric Kit (BioVision, Milpitas, CA, USA). Insulin-like growth factor-1 (IGF-1) was evaluated in tissue homogenates using the Rabbit Insulin-like Growth Factor 1 ELISA Kit (Cusabio Technology, Houston, TX, USa).

### Ethical committee and Ministry of Health approval

Animal handling complied with Animal Welfare Body of the University of Florence, Florence, Italy, in accordance to the Italian Ministerial Law n. 26/2014. The study complied with the Ministry of Health authorization n. 602/2020-PR.

### Liver histomorphology

Liver specimens were fixed in 10% buffered formalin, paraffin embedded and sectioned at a thickness of 5 μm with a microtome. Slides were then analyzed to evaluate inflammation and lipid accumulation utilizing Giemsa and Masson’s trichrome staining, respectively. Briefly, deparaffinized and rehydrated sections were incubated with Giemsa (Bio-Optica, Milan, Italy) in distilled water at ratio 1:1 or with Masson’s trichrome (Bio-Optica), following the manufacturer’s instructions, as previously described [24].

Collagen content evaluation for fibrosis grade and quantification in liver was carried out by staining using Picrosirius Red Stain kit (Bio-Optica) per the manufacturer's instructions. Briefly, deparaffinized and rehydrated sections were incubated with the staining solution for 50 min, rinsed with the appropriate reagents and with water, dehydrated through ascending alcohols and xylene, and mounted, as previously described [34].

Fibrosis grade was scored by Ishak scoring as: 0 = no fibrosis; 1 = fibrosis in some portal areas with or without short septa; 2 = fibrosis in most portal areas with or without short septa; 3 = fibrosis in most portal areas with occasional portal to portal bridging; 4 = fibrosis in most portal areas with marked bridging (portal to portal/central); 5 = fibrosis with marked bridging (portal to portal and portal to central) and with occasional nodules; 6 = cirrhosis [35, 36].

Percentage of the sampled area was performed on 40× original magnification slides, using open source Java-based ImageJ software (Fiji bundle, downloadable at https://imagej.net/). All slides were evaluated blindly and photographed using a Nikon Microphot-FXA microscope (Nikon, Tokyo, Japan).

### RNA extraction and quantitative RT-PCR analysis

TRIzol reagent (Life Technologies, Paisley, UK) and/or RNeasy Mini Kit (Qiagen, Hilden, Germany) were used to isolate total RNA from rabbit liver specimens. cDNA synthesis was carried out using the iScript™ cDNA Synthesis Kit (Bio-Rad Laboratories, Hercules, CA, USA). Quantitative real-time RT-PCR (qRT-PCR) amplification and detection was performed with SsoAdvanced™ Universal SYBR^®^ Supermix and a CFX96 Two-Color Real-Time PCR Detection System (both Bio-Rad Laboratories).

Specific PCR primers for rabbit target genes were designed on sequences available at the National Center for Biotechnology Information GenBank (http://www.ncbi.nlm.nih.gov) or Ensemble Genome (http://www.ensembl.org). The 18S ribosomal RNA subunit was evaluated with a predeveloped assay (Hs99999901_s1; Life Technologies) and used as the reference gene for the relative quantization of the target genes based on the comparative threshold cycle (Ct) 2^−ΔΔCt^ method [37].

### Statistical analysis

Results are expressed either as mean or fold-change arbitrary units ± standard error of the mean (SEM). The statistical analysis was carried out with a one-way ANOVA Kruskal–Wallis non-parametric test followed by Mann–Whitney post-hoc analysis to evaluate differences between groups, with *p* < 0.05 considered as significant.

Where applicable, Pearson’s chi-square and Fisher’s exact tests were used for contingency tables (i.e. scoring of nominal values), whereas Spearman’s test was used for correlation analysis. All statistical analyses were performed with software package SPSS 26.0 (SPSS Inc., Chicago, IL, USA).

## Results

### Visceral appearance of sacrificed animals

**Fig. 1:**
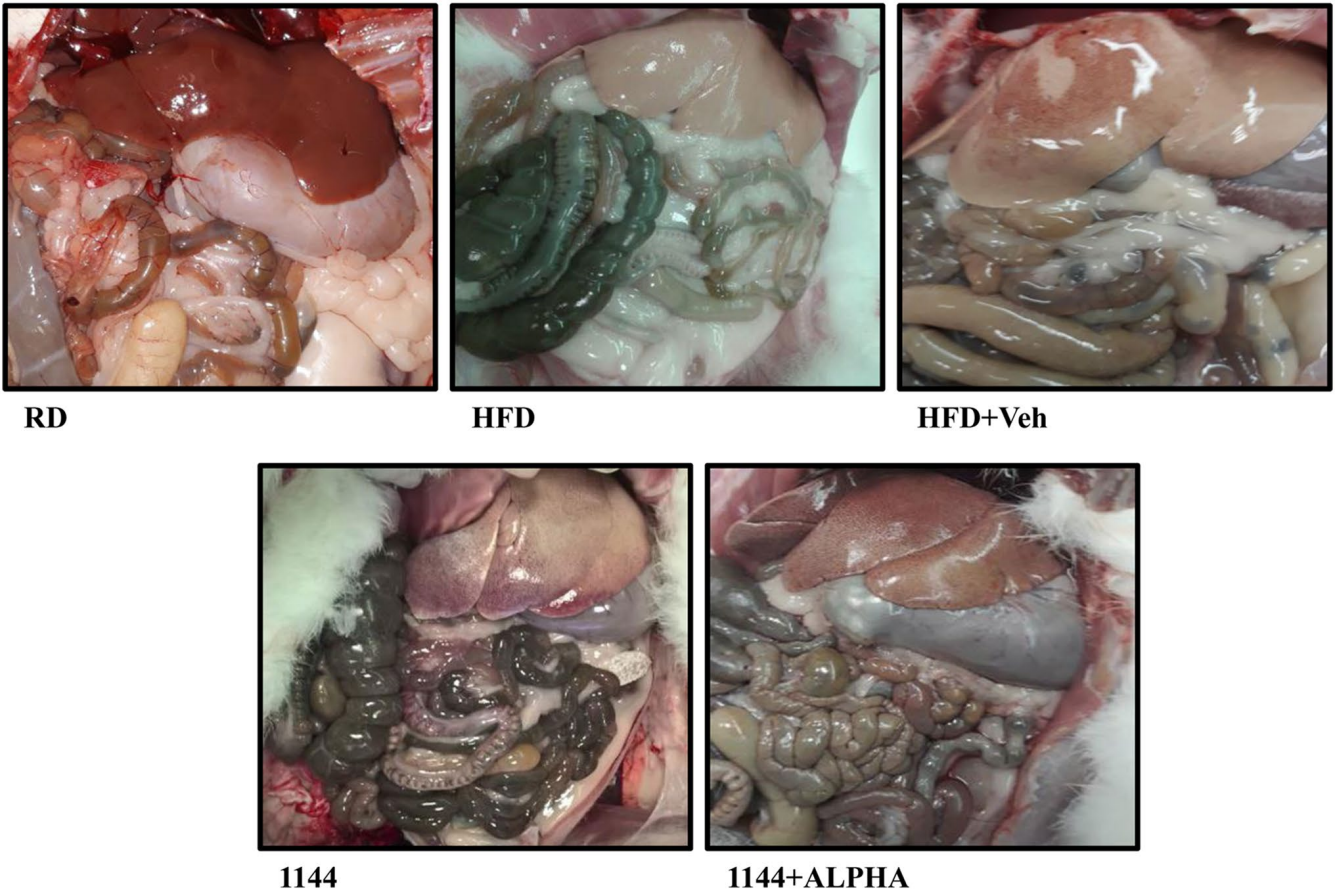
Representative images of dissected carcasses from each experimental group

Figure 1 shows the abdominal cavity of representative animals. The images clearly display the typical macroscopic features of a steatotic liver in all HFD groups, including 1144 treatment groups showing only mildly counterbalancing the accumulation of hepatic fat. HFD groups without 1144 treatment (HFD and HFD + Veh groups) also showed significantly increased visceral fat accumulation. It is noteworthy that 1144 with or without α-Tocopherol, dramatically reduced the HFD-induced visceral adiposity.

### Effects of treatments on biochemical analyses

Table 2 reports the biochemical data obtained from RD (*n* = 10), HFD (*n* = 10), HFD + Veh (*n* = 7), 1144 (*n* = 8) and 1144 + ALPHA (*n* = 8) rabbits. All data were compared to those obtained in the RD group. As previously described, feeding a HFD for 12 weeks was able to induce MetS features, including significant increase of glycaemia, MAP, cholesterol and triglyceride levels, as well as of visceral adiposity (expressed as percentage of total body weight), when compared to RD animals (Table 2). These changes were associated with a significant reduction of total testosterone, coupled with a significant weight decrease of the two most androgen-dependent organs, namely prostate and seminal vesicles (calculated as percentage of total body weight). As shown in Table 2, male rabbits fed HFD and HFD + Veh for 12 weeks showed a significantly greater area under the curve of plasma glucose during OGTT, thus demonstrating a reduced glucose tolerance. HFD and HFD + Veh rabbits also showed a significant increase in plasma ALP, GGT, bilirubin, AST and ALT, when compared to RD rabbits, as well as a significant increase in liver triglycerides content and an augmented liver weight (expressed as percentage of total body weight) (Table 2). No relevant differences were observed between the HFD and HFD + Veh groups in the parameters analyzed, thereby indicating that the vehicle formulation does not affect the overall MetS phenotype (Table 2).

**Table 2:** Metabolic parameters in the experimental rabbits from all groups

Analysis	RD (*n* = 10)	HFD (*n* = 10)	HFD + Veh (*n* = 7)	1144 (*n* = 8)	1144 + ALPHA (*n* = 8)
Total body weight (g)	3,896.70 ± 39.97	3,676.00 ± 50.59**	3,577.14 ± 64.81**	3,377.13 ± 73.38**^,^°°	3,299.13 ± 35.97***^,^°°^,çç^
Glycaemia (g/L)	1.01 ± 0.05	1.84 ± 0.28**	1.40 ± 0.14*	1.18 ± 0.05*^,^°	1.08 ± 0.14°
OGTT (iAUC)	143.21 ± 7.06	227.04 ± 19.78**	209.70 ± 21.56**	180.04 ± 5.23**^,^°	156.65 ± 8.36°°^,ç,#^
Cholesterol (mg/dL)	35.10 ± 4.26	1,847.20 ± 234.43***	1,651.29 ± 211.89**	2,045.13 ± 184.33***	1,682.88 ± 155.90***
Triglycerides (mg/dL)	72.40 ± 11.03	237.60 ± 49.40**	177.43 ± 17.59**	123.25 ± 9.28**^,ç^	149.98 ± 18.50**
Triglycerides (nmol/mg liver)	8.70 ± 0.50	18.18 ± 0.88***	17.85 ± 1.16**	15.43 ± 0.82***^,^°	15.90 ± 1.46***
ALP (U/L)	42.50 ± 3.64	74.80 ± 5.87***	65.00 ± 2.50**	52.19 ± 4.87°	87.50 ± 18.67**
GGT (U/L)	10.85 ± 1.21	82.20 ± 25.18***	63.00 ± 23.74*	129.50 ± 43.62**	236.13 ± 46.18***^,^°^,çç^
Total bilirubin (µmol/L)	2.89 ± 0.80	40.86 ± 11.40**	37.00 ± 17.49*	4.16 ± 1.07°°	7.79 ± 1.53*^,^°
AST (U/L)	40.00 ± 6.76	91.90 ± 12.98**	95.00 ± 6.55**	106.25 ± 8.11**	125.88 ± 14.49**
ALT (U/L)	35.40 ± 4.72	61.80 ± 9.18*	82.14 ± 15.44**	84.13 ± 10.02**	91.88 ± 9.77**^,^°
IGF-1 (ng/mL)	29.80 ± 3.66	15.57 ± 1.29**	20.39 ± 2.14*^,^°	26.44 ± 3.42°°	25.84 ± 3.49°°
IGF-1 (ng/mg liver)	0.84 ± 0.04	0.67 ± 0.03*	0.70 ± 0.03*	0.84 ± 0.07°	0.79 ± 0.03°
Albumin (g/L)	38.00 ± 1.36	33.10 ± 1.83*	45.29 ± 1.98**^,^°°	42.75 ± 0.80*^,^°°	43.25 ± 1.33*^,^°°
SHBG (nmol/L)	119.77 ± 3.30	97.29 ± 4.79**	107.74 ± 7.39	101.81 ± 4.01*	113.07 ± 5.27
Testosterone (nmol/L)	6.55 ± 0.94	2.89 ± 0.84**	3.26 ± 0.81*	15.35 ± 3.85°°^,çç^	11.52 ± 1.97°°^,çç^
MAP (mmHg)	86.88 ± 3.28	141.58 ± 9.60***	143.75 ± 6.85**	109.06 ± 3.15**^,^°^,çç^	107.34 ± 3.30**^,^°^,çç^
Liver weight (% of body weight)	2.66 ± 0.15	3.93 ± 0.18***	3.84 ± 0.07**	3.47 ± 0.12**^,ç^	3.13 ± 0.16*^,^°°^,çç^
VAT weight (% of body weight)	0.86 ± 0.07	1.10 ± 0.08**	1.05 ± 0.02**	0.49 ± 0.06**^,^°°°^,çç^	0.30 ± 0.04***^,^°°°^,çç,#^
Prostate weight (% of body weight)	0.018 ± 0.004	0.009 ± 0.001**	0.011 ± 0.001	0.021 ± 0.001°°°^,çç^	0.021 ± 0.002*^,^°°°^,çç^
Seminal vesicles weight (% of body weight)	0.019 ± 0.001	0.012 ± 0.001**	0.012 ± 0.001**	0.025 ± 0.002*^,^°°°^,çç^	0.024 ± 0.002°°°^,çç^

Results are reported as mean ± SEM. All biomarkers resulted statistically significant at ANOVA one-way Kruskal–Wallis analysis. These biomarkers were further analyzed by Mann–Whitney test to evaluate statistical differences between single groups

*iAUC* incremental area under the curve of glucose blood level during oral glucose tolerance test (OGTT), *ALP* alkaline phosphatase, *GGT* gamma-glutamyl transferase, *AST* aspartate aminotransferase, *ALT* alanine aminotransferase, *IGF-1* insulin-like growth factor 1, *SHBG* sex hormone binding globulin, *MAP* mean arterial pressure, *VAT* visceral adipose tissue

*p < 0.05, **p < 0.01, ***p < 0.001 vs. RD; °*p* < 0.05, °°*p *< 0.01, °°°*p* < 0.001 vs. HFD; ^ç^*p* < 0.05, ^çç^*p* < 0.01 vs. HFD + Veh; ^#^*p* < 0.05 vs. 1144

In 1144 and 1144 + ALPHA groups, HFD-induced increase in liver weight and its relative triglyceride content were partially counteracted by the treatments.

Treatment with either 1144 or 1144 + ALPHA also induced a sharp increase in testosterone (T) level, as compared to the HFD and HFD + Veh groups. T increase was associated with a normalization of prostate and seminal vesicles weight (calculated as percentage of total body weight, Table 2). 1144 and 1144 + ALPHA groups also showed a significant decrease in glycaemia and an improved glucose tolerance (OGTT), with a nonsignificant reduction in circulating triglycerides. Interestingly, a striking reduction in visceral fat, reaching a level that was even below that of RD group, was observed in the 1144 treated groups (Table 2).

No statistically significant changes in both 1144-treated groups were observed for cholesterol, ALP, GGT, AST and ALT, as compared to HFD groups. In contrast, circulating and hepatic IGF-1, decreased by the HFD condition, were restored by 1144 dosing. In addition, the dramatic increase in bilirubin level associated to HFD was substantially restored up to the RD level in both 1144 arms. Finally, in HFD and HFD + Veh groups we observed a mild decrease in total body weight, which was further exacerbated by 1144 or 1144 + ALPHA dosing (Table 2).

### *Effects of 1144 and 1144* + *ALPHA treatments on liver histomorphology*

**Fig. 2:**
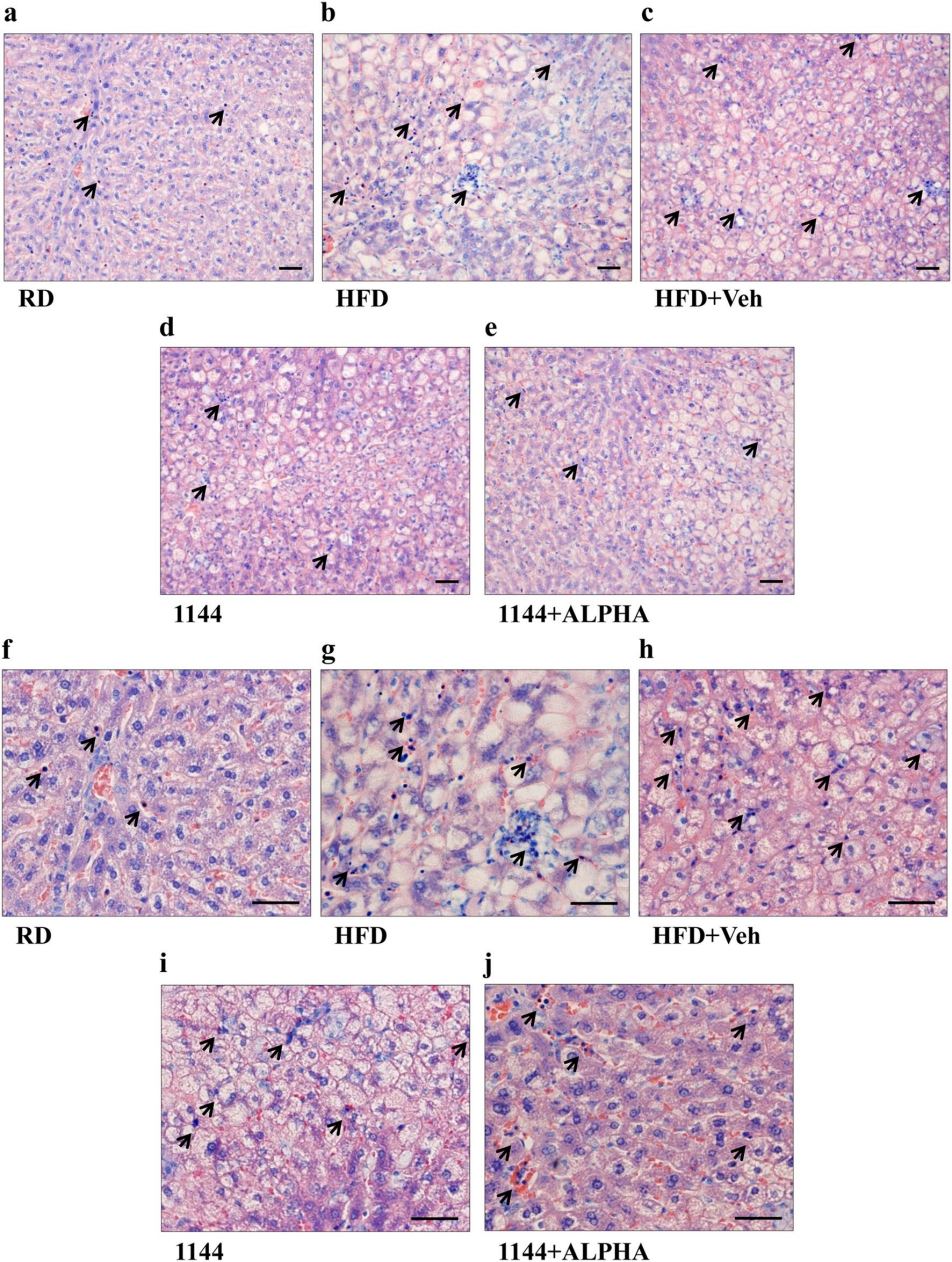
Giemsa staining of liver specimens. Representative images of RD (**a**, **f**), HFD (**b**, **g**), HFD + Veh (**c**, **h**), 1144 (**d**, **i**), and 1144 + ALPHA (**e**, **j**) samples at ×100 and ×200 original magnifications, respectively. Black arrows indicate foci of inflammatory mononuclear infiltrates (dark blue/purple nuclei). Scale bar = 50 µm

**Fig. 3:**
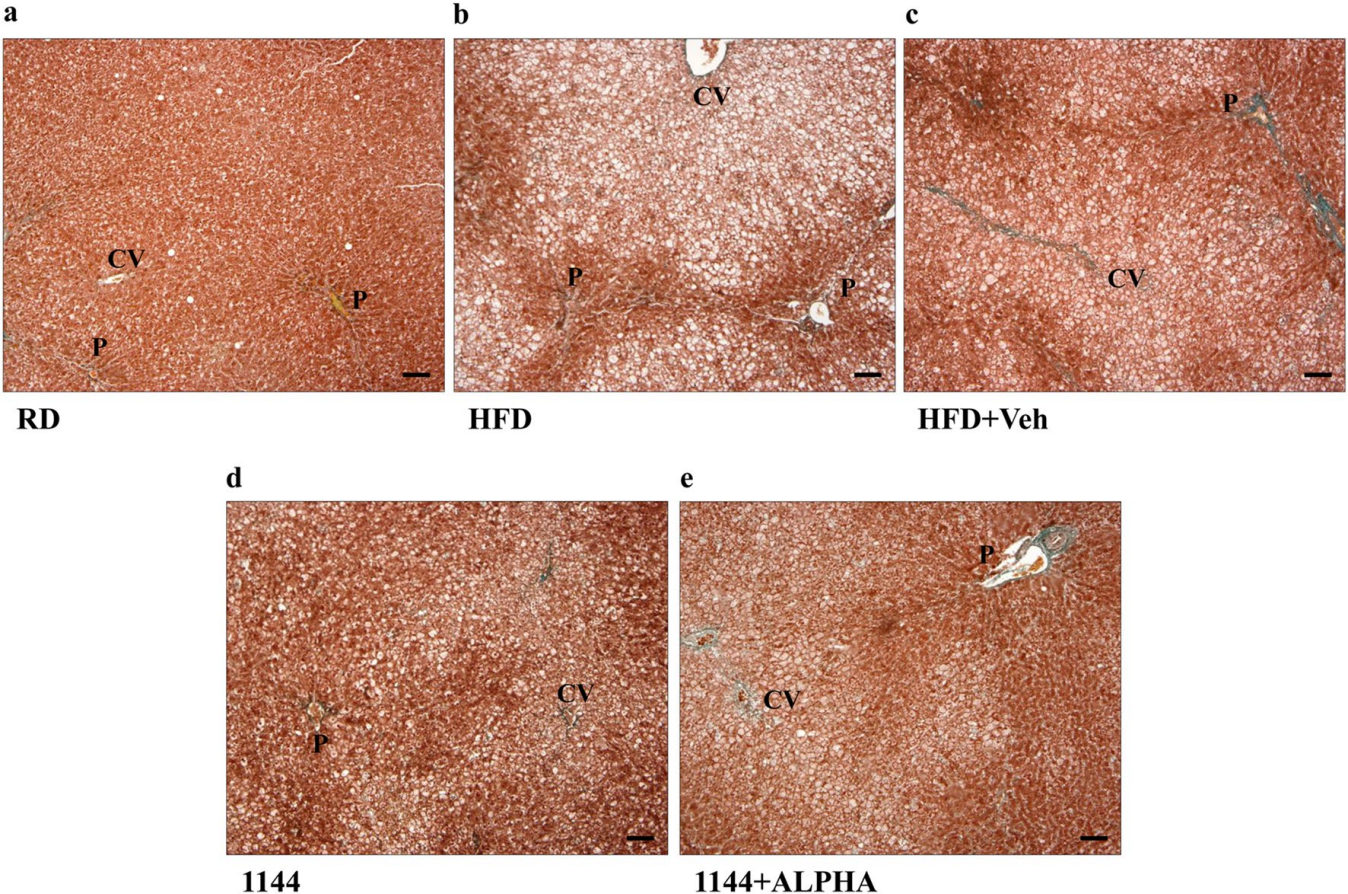
Masson’s trichrome histomorphological analysis of liver sections steatosis. **a**–**e** Show representative images of RD, HFD, HFD + Veh, 1144, and 1144 + ALPHA samples, respectively. *CV* central vein; *P *portal area. Scale bar = 100 µm

**Fig. 4:**
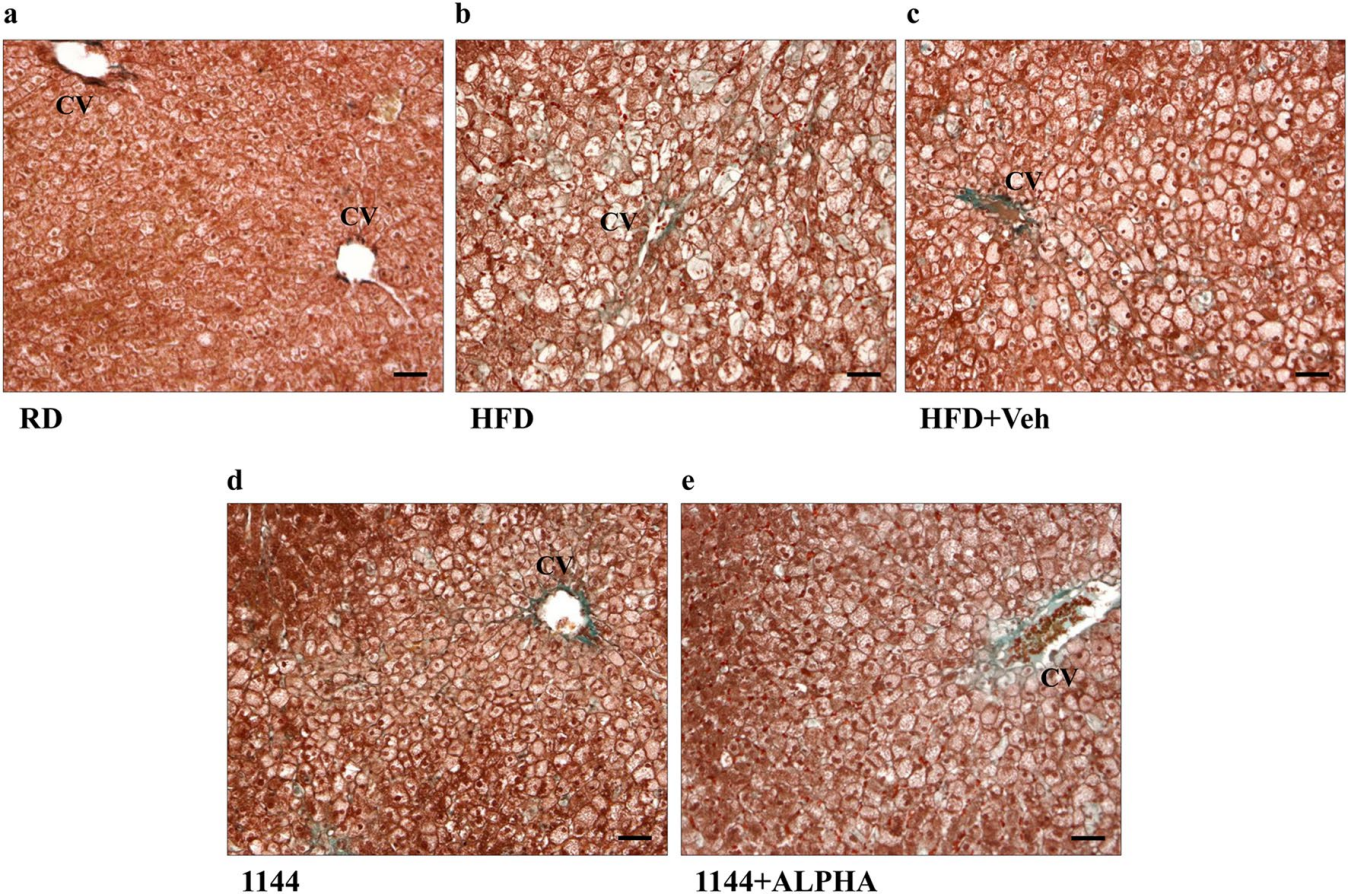
Masson’s trichrome histomorphological analysis of liver sections ballooning. **a**–**e** Show representative images of RD, HFD, HFD + Veh, 1144, and 1144 + ALPHA samples, respectively. *CV *central vein. Scale bar = 50 µm

**Fig. 5:**
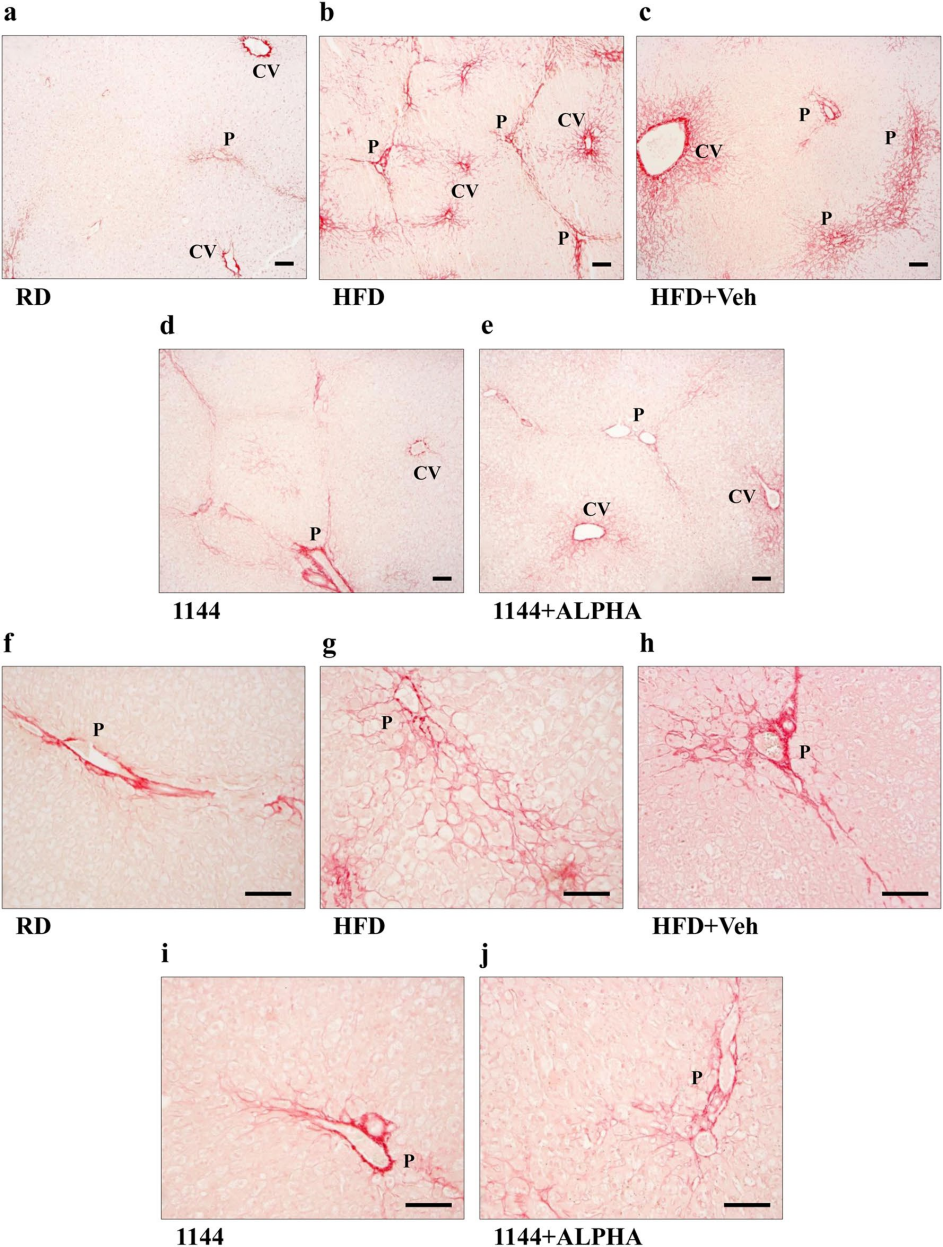
Picrosirius Red analysis of collagen deposition in rabbit liver sections. Representative images of RD (**a**, **f**), HFD (**b**, **g**), HFD + Veh (**c**, **h**), 1144 (**d**, **i**), and 1144 + ALPHA (**e**, **j**) samples at ×40 and ×100 original magnifications, respectively. *CV *central vein, *P *portal area. Scale bar = 100 µm

We first performed liver histomorphological analysis of the liver samples from the different experimental groups, assessing inflammatory infiltrates ( using Giemsa staining; Fig. 2), steatosis and hepatocyte ballooning ( using Masson’s trichrome staining; Figs. 3 and 4) and fibrosis ( using Picrosirius Red staining; Fig. 5). Figure 2 shows representative images of 100× and 200× original magnifications of Giemsa staining in the different groups. Compared to RD (Fig. 2a, f), HFD and HFD + Veh sections (Fig. 2b/g and c/h, respectively) clearly show the presence of numerous foci of inflammatory mononuclear infiltrates (dark blue/purple nuclei, black arrows). This feature is partially reduced by 1144 and by 1144 + ALPHA dosing (Fig. 2d/i and e/j, respectively).

The inflammation contingency scoring grades for each group, analyzed according to Kleiner et al. [38], are reported in Table 3. The inflammation scores recorded in the 1144 and 1144 + ALPHA groups were numerically improved as compared to HFDs, albeit without reaching statistical significance, particularly in the most severe class of inflammation (score = 3).

**Table 3:** Inflammation, steatosis, ballooning and fibrosis scores in liver extracts

Analysis	Contingency table scores	RD (*n* = 10)	HFD (*n *= 10)	*Sig*.	HFD + Veh (*n* = 7)	*Sig.*	1144 (*n* = 8)	*Sig.*	1144 + ALPHA (*n* = 8)	*Sig.*
Inflammation°	0	90%	0%	***	0%	**	0%	**	0%	**
	1	10%	10%		42.8%		37.5%		50%	
	2	0%	50%		28.6%		50%		37.5%	
	3	0%	40%		28.6%		12.5%		12.5%	
Steatosis^+^	0	100%	0%	***	0%	***	0%	***	0%	***
	1	0%	100%		100%		100%		100%	
Ballooning^	0	100%	0%	***	0%	***	0%	*** °	0%	***
	1	0%	20%		42.8%		75%		50%	
	2	0%	80%		57.2%		25% ^#^		50%	
Fibrosis (Ishak score)^#^	Median [Quartiles]	0.50 [0.00–1.00]	3.00 [2.75–4.00]	***	3.00 [2.00–3.00]	***	2.00 [2.00–3.00]	**, §	2.50 [1.25–4.00]	**
Advanced fibrosis	Ishak score ≥ 3	0%	80%^^^		71.4%^^		37.5% ^$^		50% ^	
Fibrosis (%)^Ç^	Median [Quartiles]	1.43 [1.25–1.72]	6.63 [4.47–8.90]	***	4.45 [4.38–5.52]	**	2.70 [1.60–4.44]	*^,^ °	3.20 [2.03–6.84]	** §§

Significance (*Sig.*): Pearson’s Chi Square test was used for Inflammation, Steatosis and Ballooning

*p < 0.05, **p < 0.01, ***p < 0.001 vs. RD; °*p* < 0.05, ^§^*p* = 0.082, ^§§^*p* = 0.051 vs. HFD

Fisher’s exact test: ^*p* < 0.05, ^^*p* < 0.01, ^^^*p* < 0.001 vs. RD; ^#^*p* < 0.05, ^$^*p* = 0.088 vs. HFD

°Inflammation scores are defined as follows [38]: 0 = no inflammation foci; 1 =  < 2 foci per 200 × field; 2 = 2–4 foci per 200 × field;3 =  > 4 foci per 200 × field

** + **Steatosis scores are defined as follows: 0 = no steatosis; 1 = prominent steatosis

^Ballooning scores are defined as follows [38]: 0 = no ballooning; 1 = few balloon cells; 2 = prominent ballooning

^#^Fibrosis scores are defined as follows (Ishak Score) [35, 36]: 0 = no fibrosis; 1 = fibrosis in some portal areas with or without short septa; 2 = fibrosis in most portal areas with or without short septa; 3 = fibrosis in most portal areas with occasional portal to portal bridging; 4 = fibrosis in most portal areas with marked bridging (portal to portal/central); 5 = fibrosis with marked bridging (portal to portal and portal to central) and withoccasional nodules; 6 = cirrhosis

^Ç^Fibrosis determined as percentage of sampled area using ImageJ software

The extent of steatosis within the liver of the different groups was examined by Masson’s trichrome staining. Figure 3 shows representative images at 40× original magnifications of RD (panel a), HFD (panel b), HFD + Veh (panel c), 1144 (panel d), and 1144 + ALPHA (panel e) specimens. The steatotic effects of HFD in all animals are particularly visible around the centrilobular zone. An eyeball analysis on the effect of 1144 and 1144 + ALPHA on steatosis suggest a mild reduction, however this was not confirmed by scoring, most presumably due to the binomial categorization of the scoring system. The contingency value scoring grades for each of the groups analyzed are reported in Table 3.

Similar to the evaluation of steatosis, the presence of hepatocytes ballooning was evaluated with a higher magnifications (100×) of the Masson’s trichrome staining. Figure 4 reports representative images of RD (panel a), HFD (panel b), HFD + Veh (panel c), 1144 (panel d), and 1144 + ALPHA (panel e) groups. In the centrilobular zone of HFD sections, we observed a disarranged lobular structure with most hepatocytes presenting ballooning, as compared to the normal structure and absence of lipids in RD samples. The scoring grades for each of the groups analyzed are reported in Table 3. 1144 dosing was associated with a significant improvement in the contingency table ballooning score vs. HFD (*p* < 0.05 with Pearson’s Chi Square Test), in particular reaching statistical significance in the most severe class (score = 2; *p* < 0.05 with Fisher’s Exact Test).

We then studied the effect of HFD on fibrosis by analyzing samples with Picrosirius Red staining. Figure 5 displays representative images of RD (panels a and f), HFD (panels b and g), HFD + Veh (panels c and h), 1144 (panels d and i), and 1144 + ALPHA (panels e and j) specimens. HFD sections were characterized by collagen deposition forming portal to portal bridges and, occasionally, portal to central bridges. In particular, under higher magnification, peri-cellular and sinusoidal fibrosis and the presence of a “chicken wire” pattern, typical of advanced stages of fibrosis, are apparent in HFD and HFD + Veh samples (Fig. 5g, h, respectively). These peculiar features are clearly decreased in both 1144 and 1144 + ALPHA groups (Fig. 5i, j). In the RD group, we observed only minimal changes in the physiological structural of the liver along with a mild collagen deposition (Fig. 5a, f), as already reported in other studies in rabbits [39].

Fibrosis was further investigated by observation of slides under 40× magnification and by reporting the Ishak scores and the percentage of fibrosis, the latter through densitometry of the collagen deposition. Table 3 reports the median and quartiles of the Ishak score for each group. In the HFD and HFD + Veh samples, Ishak score and percentage of fibrosis of sampled area resulted significantly increased, as compared to the RD group (Table 3). Although 1144-treated arms showed a reduced collagen deposition, according to both Ishak score and percentage of fibrosis in sampled areas, these differences reached full statistical significance only on percentage of fibrosis in 1144 arm (Table 3). The percentage of rabbits with advanced fibrosis (Ishak score ≥ 3) was numerically reduced in 1144 and 1144 + ALPHA arms, compared to HFD and HFD + Veh arms. However, these differences did not reach statistical significance (Table 3).

**Fig. 6:**
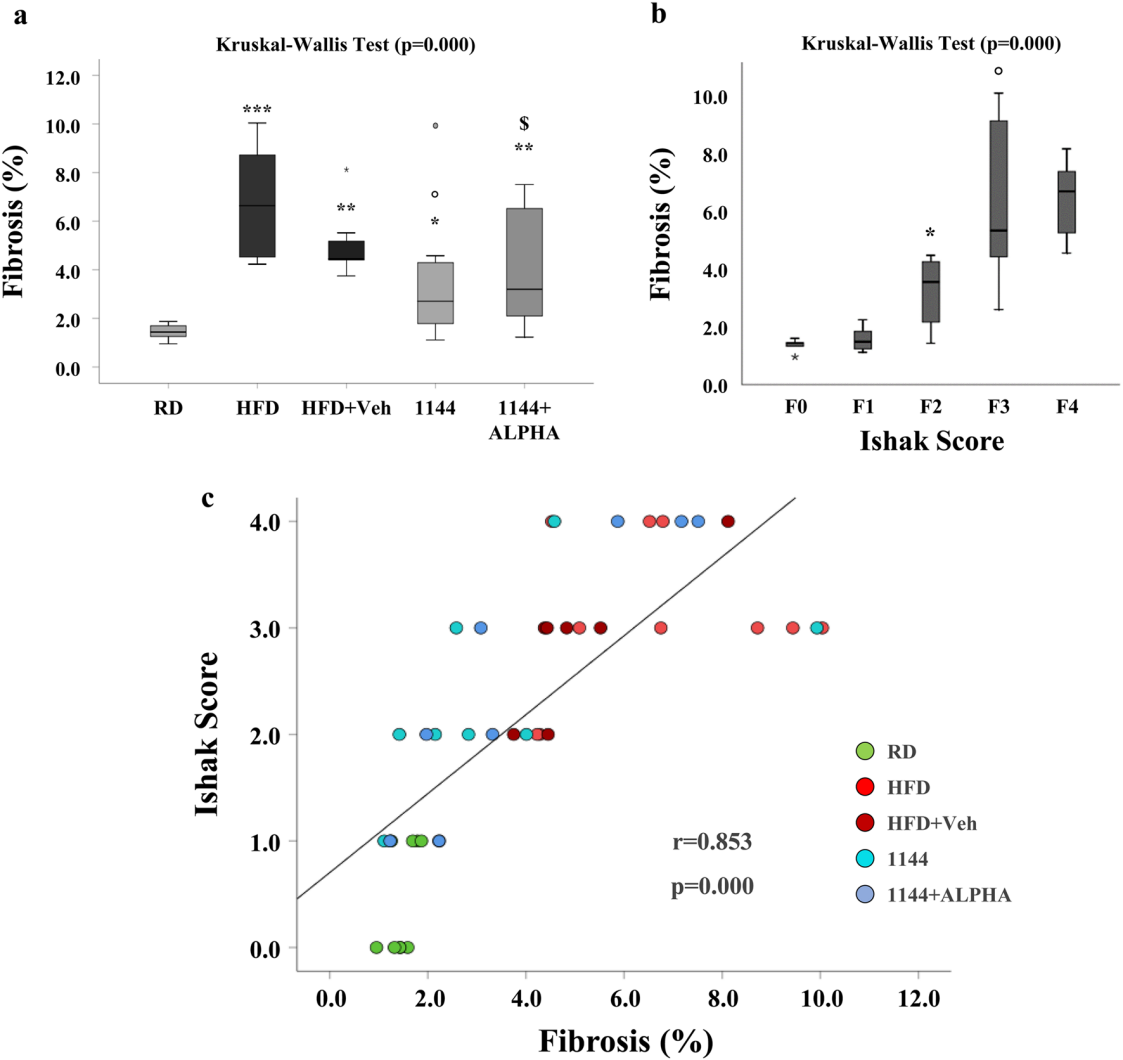
Picrosirius Red analysis of collagen deposition in rabbit liver sections. **a** Shows the percentage of collagen over the sampled area in RD, HFD, HFD + Veh, 1144 and 1144 + ALPHA groups (*p < 0.05, **p < 0.01, ***p < 0.001 vs. RD; °*p* < 0.05, ^**$**^*p* = 0.051 vs. HFD). **b** Shows the percentage of collagen over the sampled area in relation to fibrosis Ishak scores (F0 = 0, F1 = 1, F2 = 2, F3 = 3, F4 = 4; *p < 0.001 vs. F1; °*p* < 0.001 vs. F2). **c** Shows the Spearman’s correlation of fibrosis Ishak scores and percentage of sampled area (*r* = 0.853; *p* = 0.000)

Figure 6a reports the percentage of fibrosis of the sampled areas across all experimental groups as box plots. It clearly shows a significant increase in the fibrotic area in HFD and HFD + Veh groups as compared to RD group. 1144 and 1144 + ALPHA groups partially improved this feature, as compared to HFD groups, however without reaching RD level. Figure 6b shows the relationship between the progressive severity of the clinical score and the percentage of fibrosis, showing an increase in fibrosis as a function of Ishak score severity. This is further demonstrated in Fig. 6c, which reports the highly significant correlation (*r* = 0.853; *p* < 0.001) between the clinical score and the percentage of fibrosis of the sampled area in animals from all groups.

### *Effects of 1144 and 1144* + *ALPHA treatments on mRNA markers expression*

The mRNA expression of numerous genes specifically involved in the pathological process of NAFLD was significantly upregulated in the HFD and HFD + Veh animals, when compared to RD group. Treatments with either 1144 or 1144 + ALPHA significantly improved several of these HFD-induced molecular alterations (Tables 4, 5 ,6, 7). In particular, both HFD groups showed a significant increase in mRNA expression of MCP1 and TNFα, whilst both 1144 treatments significantly counteracted these increases. HFD feeding also induced a significant increase in mRNA expression of several key pro-inflammatory genes (including CD68, GATA3, LOX1, TLR2, TLR4), while the two 1144 treatments showed a clear trend toward a reduction in their expression, as well as an improvement for pro- (CD11c) and anti-inflammatory (CD206) macrophage markers (M1 and M2 subtypes, respectively) (Table 4).

**Table 4:** Inflammation genes mRNA expression (qRT-PCR) in liver extracts

Inflammatory Markers	RD (*n* = 10)	HFD (*n* = 10)	HFD + Veh (*n* = 7)	1144 (*n* = 8)	1144 + ALPHA (*n* = 8)
**CD11c**	**1.00 ± 0.20**	41.37 ± 10.78**	**18.45 ± 2.93**	31.17 ± 4.49**^,ç^	23.64 ± 4.52**
CD206	1.00 ± 0.15	0.73 ± 0.08	0.61 ± 0.05	0.85 ± 0.12	0.87 ± 0.15
**CD68**	**1.00 ± 0.13**	9.05 ± 1.77**	3.58 ± 0.43*^,^**°°**	6.13 ± 0.63**^,çç^	3.74 ± 0.65*^,^**°°**^**,#**^
**COX2**	**1.00 ± 0.12**	5.55 ± 0.99*	6.48 ± 2.77*	6.48 ± 1.54**	6.50 ± 1.40**
**GATA3**	**1.00 ± 0.20**	**1.72 ± 0.19**	**1.64 ± 0.61**	**1.27 ± 0.24**	**0.92 ± 0.14°**
IL1β	1.00 ± 0.27	1.75 ± 0.38	1.89 ± 0.35	1.90 ± 0.35	1.60 ± 0.45
IL6	1.00 ± 0.14	2.97 ± 1.11	3.42 ± 0.67	1.65 ± 0.34	1.42 ± 0.30
**IL8**	**1.00 ± 0.17**	22.49 ± 5.09**	28.98 ± 6.66*	20.89 ± 2.44**	15.76 ± 2.72**
**IL10**	**1.00 ± 0.10**	13.40 ± 1.85**	8.34 ± 0.41*^,^**°**	9.83 ± 1.71**	7.20 ± 0.79**^,^**°**
IL12p35	1.00 ± 0.23	1.14 ± 0.25	1.75 ± 0.16	0.68 ± 0.15	0.83 ± 0.15
**IL12p40**	**1.00 ± 0.06**	**0.69 ± 0.11**	0.63 ± 0.08*	0.48 ± 0.06*	0.41 ± 0.06**
**LOX1**	**1.00 ± 0.09**	14.85 ± 3.81*	11.17 ± 3.17*	7.05 ± 1.46**	5.66 ± 1.32**^,ç^
**MCP1**	**1.00 ± 0.23**	32.22 ± 9.00**	9.52 ± 1.33*	4.51 ± 0.93*^,^**°°**^**,ç**^	**3.38 ± 0.96°°**^**,çç**^
RAGE	1.00 ± 0.22	1.59 ± 0.26	1.84 ± 0.21	1.24 ± 0.16	1.22 ± 0.13
RORγt	1.00 ± 0.24	0.87 ± 0.22	0.69 ± 0.21	0.80 ± 0.13	0.80 ± 0.11
**TBET**	**1.00 ± 0.17**	2.12 ± 0.24*	2.11 ± 0.44*	**1.56 ± 0.21**	**1.55 ± 0.24**^**ç**^
**TLR2**	**1.00 ± 0.08**	9.77 ± 1.69**	4.51 ± 0.51*^,^**°°**	3.76 ± 0.67*^,^**°°**	4.33 ± 1.00**^,^**°**
**TLR4**	**1.00 ± 0.14**	3.44 ± 0.48*	3.97 ± 0.90*	2.78 ± 0.35*	2.02 ± 0.30*^,^**°**
**TNFα**	**1.00 ± 0.19**	5.08 ± 0.82**	3.31 ± 0.34*	2.39 ± 0.35*^,^**°**^**,ç**^	**1.99 ± 0.33°°**^**,**^**ç**

Results are expressed as fold-change vs RD and are reported as mean ± SEM. In bold are reported the genes that resulted statistically significant at ANOVA one-way Kruskal–Wallis analysis. These genes were further analyzed by Mann–Whitney test to evaluate statistical differences between single groups. No further tests were performed for those genes that did not show statistical differences at ANOVA one-way Kruskal–Wallis

*CD11c* integrin, alpha X, *CD206*, cluster of differentiation 206; *CD68* cluster of differentiation 68, *COX2* inducible cyclooxygenase-2, *GATA3* Th2 lymphocytes transcription factor, *IL1β* interleukin 1 subunit beta, *IL6* interleukin 6, *IL8* interleukin 8, *IL10* interleukin 10, *IL12p35* interleukin-12 subunit p35, *IL12p40* interleukin-12 subunit p40, *LOX1* lectin-type oxidized LDL receptor 1, *MCP1* monocyte chemoattractant protein-1, *RAGE* receptor for advanced glycation endproducts, *RORγt* RAR-related orphan receptor gamma, *TBET* T-box transcription factor TBX21, *TLR2* toll-like receptor 2, *TLR4* toll-like receptor 4, *TNFα* tumor necrosis factor alpha

*p < 0.01, **p < 0.001 vs. RD; °*p* < 0.05, °°*p* < 0.01 vs. HFD; ^ç^*p* < 0.05, ^çç^*p* < 0.01 vs. HFD + Veh; ^#^*p* < 0.05 vs. 1144

**Table 5:** Fibrosis genes mRNA expression (qRT-PCR) in liver extracts

Fibrosis markers	RD (*n* = 10)	HFD (*n* = 10)	HFD + Veh (*n* = 7)	1144 (*n* = 8)	1144 + ALPHA (*n* = 8)
**COL1A1**	**1.00 ± 0.16**	21.03 ± 5.86**	11.90 ± 3.40*	8.91 ± 1.90**	10.82 ± 2.08*
**COL3A1**	**1.00 ± 0.17**	7.40 ± 2.10**	**3.90 ± 0.97**	3.44 ± 0.77*	**3.96 ± 0.98**
ET1	1.00 ± 0.08	1.36 ± 0.09	1.20 ± 0.11	1.07 ± 0.08	1.14 ± 0.16
**ETRA**	**1.00 ± 0.10**	2.93 ± 0.55**	**1.19 ± 0.18°°**	**2.01 ± 0.40**	**1.62 ± 0.36**
**ETRB**	**1.00 ± 0.08**	2.59 ± 0.28**	**1.33 ± 0.13°°**	**1.48 ± 0.19°°**	**1.18 ± 0.21°°**
FN1	1.00 ± 0.08	1.10 ± 0.14	1.11 ± 0.13	0.97 ± 0.15	0.81 ± 0.13
**FOXP3**	**1.00 ± 0.14**	3.90 ± 0.80**	2.50 ± 0.48*	**1.56 ± 0.22°**	**1.62 ± 0.23°**
**MMP2**	**1.00 ± 0.15**	18.47 ± 8.05**	11.82 ± 2.60*	25.20 ± 5.14**	26.31 ± 9.32**
**MMP9**	**1.00 ± 0.08**	8.28 ± 1.71**	4.92 ± 0.85*	10.03 ± 2.00**^,ç^	6.90 ± 1.27**
**αSMA**	**1.00 ± 0.06**	3.60 ± 0.76**	2.48 ± 0.32*	**1.84 ± 0.44°**	**1.93 ± 0.37°**
**SNAI1**	**1.00 ± 0.30**	**2.79 ± 0.39**	4.59 ± 1.06*	**2.15 ± 0.58**	**2.20 ± 0.88**
SNAI2	1.00 ± 0.19	1.63 ± 0.24	2.03 ± 0.26	1.39 ± 0.19	1.27 ± 0.15
**TGFβ1**	**1.00 ± 0.13**	3.55 ± 0.44**	2.33 ± 0.16*^,^**°**	2.60 ± 0.33*	**1.94 ± 0.32°**
**TIMP1**	**1.00 ± 0.13**	10.69 ± 2.50**	6.88 ± 1.23*	4.63 ± 0.71**^,^**°**	4.18 ± 0.66*^,^**°°**^**,ç**^
**TIMP2**	**1.00 ± 0.08**	7.02 ± 0.75**	4.48 ± 0.86*^,^**°°**	4.91 ± 0.51**^,^**°**	3.67 ± 0.69*^,^**°°**

Results are expressed as fold-change vs RD and are reported as mean ± SEM. In bold are reported the genes that resulted statistically significant at ANOVA one-way Kruskal–Wallis analysis. These genes were further analyzed by Mann–Whitney test to evaluate statistical differences between single groups. No further tests were performed for those genes that did not show statistical differences at ANOVA one-way Kruskal–Wallis

*COL1A1* collagen type I alpha 1, COiL3A1 collagen type III alpha 1, *ET1* endothelin 1, *ETRA* endothelin receptor type A, *ETRB* endothelin receptor type B, *FN1* fibronectin 1, *FOXP3* forkhead box P3, *MMP2* matrix metalloproteinase-2, *MMP9* matrix metalloproteinase-9, *αSMA* alpha smooth muscle actin, *SNAI1* snail family transcriptional repressor 1, *SNAI2* snail family transcriptional repressor 2, *TGFβ1* transforming growth factor beta 1, *TIMP1* TIMP metallopeptidase inhibitor 1, *TIMP2* TIMP metallopeptidase inhibitor 2

*p < 0.01, **p < 0.001 vs. RD; °*p* < 0.05, °°*p* < 0.01 vs. HFD; ^ç^*p* < 0.05 vs. HFD + Veh

**Table 6:** Mitochondria biogenesis and function genes mRNA expression (qRT-PCR) in liver extracts

Mitochondria biogenesis and function markers	RD (*n* = 10)	HFD (*n* = 10)	HFD + Veh (*n* = 7)	1144 (*n* = 8)	1144 + ALPHA (*n* = 8)
FIS1	1.00 ± 0.19	1.09 ± 0.08	0.87 ± 0.12	0.93 ± 0.14	1.01 ± 0.14
MFN1	1.00 ± 0.17	1.32 ± 0.13	0.89 ± 0.06	1.03 ± 0.10	1.07 ± 0.12
MFN2	1.00 ± 0.25	1.03 ± 0.15	1.21 ± 0.27	1.05 ± 0.14	1.06 ± 0.17
NRF1	1.00 ± 0.14	1.08 ± 0.10	1.17 ± 0.18	1.08 ± 0.11	0.90 ± 0.12
OPA1	1.00 ± 0.24	0.97 ± 0.10	0.65 ± 0.05	1.06 ± 0.12	0.92 ± 0.17
PGC1α	1.00 ± 0.14	1.23 ± 0.12	1.12 ± 0.18	1.33 ± 0.22	1.81 ± 0.46
**PGC1β**	**1.00 ± 0.13**	1.52 ± 0.17*	**1.19 ± 0.21**	2.13 ± 0.26**^,ç^	**1.28 ± 0.16**^**#**^
SDHB	1.00 ± 0.22	1.15 ± 0.15	0.82 ± 0.09	1.20 ± 0.19	0.80 ± 0.17
SLC25A12	1.00 ± 0.17	1.81 ± 0.24	1.09 ± 0.15	1.73 ± 0.26	1.59 ± 0.26
TFAM	1.00 ± 0.15	0.96 ± 0.13	0.75 ± 0.07	0.79 ± 0.09	0.70 ± 0.09
UCP1	1.00 ± 0.11	0.92 ± 0.20	0.82 ± 0.15	1.08 ± 0.23	1.01 ± 0.13

Results are expressed as fold-change vs RD and are reported as mean ± SEM. In bold are reported the genes that resulted statistically significant at ANOVA one-way Kruskal–Wallis analysis. These genes were further analyzed by Mann–Whitney test to evaluate statistical differences between single groups. No further tests were performed for those genes that did not show statistical differences at ANOVA one-way Kruskal–Wallis

*FIS1* mitochondrial fission 1 protein, *MFN1* mitofusin-1, *MFN2* mitofusin-2, *NRF1* nuclear respiratory factor 1, *OPA1* mitochondrial dynamin like GTPase, *PGC1α* PPARγ coactivator 1-alpha, *PGC1β* PPARγ coactivator 1-beta, *SDHB* succinate dehydrogenase, mitochondrial, *SLC25A12* solute carrier family 25 member 12, *TFAM* mitochondrial transcription factor A, *UCP1* uncoupling protein 1

*p < 0.01, **p < 0.001 vs. RD; °*p* < 0.05, °°*p* < 0.01 vs. HFD; ^ç^*p* < 0.05, ^çç^*p* < 0.01 vs. HFD + Veh; ^#^*p* < 0.05 vs. HFD + 1144

**Table 7:** Insulin signaling and lipid handling and metabolism genes mRNA expression (qRT-PCR) in liver extracts

Insulin signaling markers	RD (*n* = 10)	HFD (*n* = 10)	HFD + Veh (*n* = 7)	1144 (*n* = 8)	1144 + ALPHA (*n* = 8)
GLUT4	1.00 ± 0.17	1.28 ± 0.23	1.14 ± 0.26	1.42 ± 0.27	1.24 ± 0.22
IRS1	1.00 ± 0.09	1.36 ± 0.24	1.32 ± 0.11	1.73 ± 0.16	1.50 ± 0.21
**STAMP2**	**1.00 ± 0.15**	**2.36 ± 0.68**	2.53 ± 1.08*	2.40 ± 0.40**	1.83 ± 0.23*
Lipid handling markers					
PLIN1	1.00 ± 0.22	1.29 ± 0.23	1.02 ± 0.13	2.52 ± 0.47	1.74 ± 0.46
SNAP23	1.00 ± 0.11	0.88 ± 0.08	0.90 ± 0.06	1.35 ± 0.22	1.19 ± 0.24
STX5	1.00 ± 0.11	1.12 ± 0.12	0.89 ± 0.08	1.12 ± 0.17	1.06 ± 0.16
VAMP4	1.00 ± 0.09	0.86 ± 0.07	1.04 ± 0.14	1.18 ± 0.10	0.96 ± 0.10
Lipid and intermediate metabolism markers					
ADPN	1.00 ± 0.20	2.70 ± 0.91	1.98 ± 0.50	1.53 ± 0.23	1.56 ± 0.27
CD36	1.00 ± 0.12	1.34 ± 0.17	1.02 ± 0.15	1.35 ± 0.15	1.42 ± 0.25
**DGAT2**	**1.00 ± 0.15**	0.29 ± 0.05***	0.20 ± 0.02**	0.33 ± 0.05**^,ç^	0.38 ± 0.08**^,ç^
**LPL**	**1.00 ± 0.23**	43.17 ± 5.88***	20.16 ± 4.95**^,^**°**	24.63 ± 6.04***^,^**°**	9.42 ± 1.59***^,^**°°**^**,#**^
PLPA2	1.00 ± 0.09	1.54 ± 0.18	1.21 ± 0.05	1.33 ± 0.12	1.15 ± 0.18
**PPARα**	**1.00 ± 0.08**	0.58 ± 0.11**	0.56 ± 0.07**	**1.06 ± 0.12°°**^**,çç**^	**0.83 ± 0.16**
**PPARγ**	**1.00 ± 0.11**	2.22 ± 0.32***	**1.37 ± 0.15°°**	2.01 ± 0.24**^,ç^	**1.57 ± 0.22**
SREBP1	1.00 ± 0.11	2.05 ± 0.43	1.15 ± 0.12	1.56 ± 0.22	1.47 ± 0.33
SREBP2	1.00 ± 0.13	1.22 ± 0.19	1.35 ± 0.24	1.10 ± 0.29	1.67 ± 0.36
**IGF-1**	**1.00 ± 0.07**	0.60 ± 0.06**	0.40 ± 0.06**	**0.86 ± 0.15**^**ç**^	**0.81 ± 0.16**^**ç**^
AR	1.00 ± 0.06	0.90 ± 0.14	0.89 ± 0.13	0.77 ± 0.14	0.68 ± 0.11

Results are expressed as fold-change vs RD and are reported as mean ± SEM. In bold are reported the genes that resulted statistically significant at ANOVA one-way Kruskal–Wallis analysis. These genes were further analyzed by Mann–Whitney test to evaluate statistical differences between single groups. No further tests were performed for those genes that did not show statistical differences at ANOVA one-way Kruskal–Wallis

*GLUT4* glucose transporter type 4, *IRS1* insulin receptor substrate 1, *STAMP2* six transmembrane protein of prostate 2, *PLIN1* perilipin 1, *SNAP23* synaptosomal-associated protein 23, *STX5* syntaxin 5, *VAMP4* vesicle-associated membrane protein 4, *ADPN* adiponectin, *CD36* cluster of differentiation 36, *DGAT2* diacylglycerol O-acyltransferase 2, *LPL* lipoprotein lipase, *PLPA2* phospholipase A2, *PPARα* perossisome proliferator-activated receptor α, *PPARγ* perossisome proliferator-activated receptor γ, *SREBP1* sterol regulatory element-binding factor 1, *SREBP2* sterol regulatory element-binding factor 2, *IGF-1* insulin-like growth factor 1, *AR* androgen receptor

*p < 0.01, **p < 0.001 vs. RD; °*p* < 0.05, °°*p* < 0.01 vs. HFD; ^ç^*p* < 0.05, ^çç^*p* < 0.01 vs. HFD + Veh; ^**#**^*p* < 0.05 vs. HFD + 1144

A number of pro-fibrotic genes (e.g. COL1A1, COL3A1, ETRs, FOXP3, αSMA, SNAI1, TGFβ1, TIMPs) were significantly overexpressed by HFD when compared to RD group, whereas 1144 and 1144 + ALPHA treatments showed a reduction or a tendency to a reduction in their mRNA expression (Table 5).

No relevant differences were observed concerning the vast majority of markers of mitochondria biogenesis and function (Table 6), the insulin signaling and the lipid handling (Table 7).

As far as the markers of lipid and intermediate metabolism are concerned, we found several significant differences, some of which were reversed by 1144 dosing (Table 7). The most impressive change is in the gene expression of LPL. LPL is known to have an adipogenic role in the NAFLD liver; as expected, mRNA expression of LPL was increased by 20–40 fold in HFD groups, as compared to RD group. Both 1144 treatments showed a significant reduction in LPL mRNA expression.

Remarkably, the mRNA expression of PPARα (a transcriptional factor related to fatty acids oxidation) and IGF-1 (inversely associated with liver damage severity) was significantly altered in HFD and HFD + Veh groups. Decreased PPARα mRNA expression was normalized by the 1144 treatment (*p* < 0.01 vs. both HFD and HFD + Veh groups), whereas IGF-1 reduction showed a marked shift towards normalization (*p* < 0.05 vs. HFD + Veh group, Table 5). Treatment with 1144 + ALPHA displayed similar trends, albeit to a lower extent.

## Discussion

Treatment with LPCN 1144 (an oral testosterone preparation), either alone or in combination with α-Tocopherol, significantly improved not only key MetS features (e.g., glycaemia, OGTT, and visceral fat accumulation), but also NASH features, including liver inflammation, steatosis, ballooning, and fibrosis. A reduction of liver triglycerides content was also observed in 1144-treated groups.

In a similar manner, as previously reported with injectable testosterone [27], treatments with 1144, and its combination with α-Tocopherol, are effective in significantly reducing glycaemia and improving insulin sensitivity (as assessed by OGTT), as well as normalizing NASH components (the hepatic hallmark of insulin resistance in MetS condition), as compared to HFDs. A general improvement on NASH was demonstrated by liver histomorphological analysis and mRNA expression analysis of specific markers of inflammatory infiltrates, steatosis and fibrosis. Using the Pearson’s Chi Square contingency tables for statistical approach, we showed that a trend toward improvement by 1144 arms was present in HFD-induced liver inflammation, steatosis and ballooning, as indicated by stratification of the data.

It is noteworthy considering the recommended clinical NASH endpoints in the clinical study guidance for treatments of NASH with fibrosis (https://www.fda.gov/regulatory-information/search-fda-guidance-documents/noncirrhotic-nonalcoholic-steatohepatitis-liver-fibrosis-developing-drugs-treatment).

In the guidance, the histological endpoints reasonably represent to predict clinical benefit are either (1) steatohepatitis resolution (NAS score of 0–1 for inflammation, 0 for ballooning, and any value for steatosis) and no worsening of fibrosis, (2) improvement in liver fibrosis and no worsening of steatohepatitis (no increase of steatosis, ballooning, or inflammation), or (3) both resolution of steatohepatitis and improvement in fibrosis.

With regard to assessment of the current results with recommended clinical NASH endpoints, hepatocyte inflammation, steatosis, and ballooning were improved in both 1144 treatment groups compared to HFD and HFD + Veh groups. As another evidence for not worsening NASH features, reduction of key mRNA pro-inflammation markers measured from liver tissues in 1144 groups, compared to HFD groups, suggests potential histological improvement of inflammation and ballooning with 1144 treatment.

In particular, the single treatment with 1144 displays a significant effect on percentage of fibrosis of sampled areas, which is further substantiated by the qualitative (clinical) Ishak score analysis. Although not as effective as 1144, the combined treatment (1144 + ALPHA) showed a borderline reduction of fibrosis. A highly significant correlation (using Spearman’s test) was observed between the Ishak score and the percentage of fibrosis of the sampled area across all experimental groups, further corroborating the finding.

The results of this study indicate that LPCN 1144 improves fibrosis without worsening of steatohepatitis (e.g., inflammation and ballooning) and prevents advancing of the NASH process. The improvement with LPCN 1144 in this model should be therefore considered as potential of clinical benefit per NASH, in accordance with the FDA guidance document. With regard to fibrosis markers, the oral androgen treatments tend to normalize/reduce the mRNA expression of genes classically involved in promoting fibrosis development (COL1A1, COL3A1, αSMA, SNAI1, TGFβ1 and the MMPs/TIMPs balance), or in the immune response linked to fibrosis (FOXP3). Especially, the LPCN 1144 treatment effects were observed for key fibrogenic factors, namely SNAI1 and TGFβ1, which are paramount to the epithelial-to-mesenchymal cell transition (EMT), a process whereby fully differentiated epithelial cells gradually switch into a mesenchymal phenotype [40]. During EMT, the TGFβ1-dependent transcription factors, SNAI1 and SNAI2, orchestrate several events including remodeling of epithelial cell–cell and cell–matrix adhesion contacts and reorganization of the actin cytoskeleton. Furthermore, αSMA mRNA expression was found to be significantly reduced in both 1144 and 1144 + ALPHA groups. This is associated with preventing the worsening of fibrosis since αSMA is a well-validated marker of stellate cell activation and fibrosis progression [41].

Both 1144 and 1144 + ALPHA treatments also demonstrated promising effects on inflammation and steatosis mRNA markers. In particular, both 1144 treatments resulted in a significant reduction in the expression of LOX1, MCP1, TLR2 and importantly, TNFα, a crucial player in the establishment of an inflammatory environment and likely a pivotal substrate for the molecular mechanism of action of testosterone. TNFα is involved in the activation of JNK and IKKβ/NF-kB pathways, toll-like receptors (TLRs) and the receptor for advanced glycation end products (RAGE), thus triggering the onset of insulin resistance in visceral fat [42]. The results with 1144 treatments are consistent with a previous study reporting that testosterone treatment inhibits JNK, IKKβ and TNFα, thus suggesting a protective mechanism of testosterone against inflammation-induced insulin resistance [43]. Numerical changes of other inflammation markers (e.g. CD11c and CD206) also provide indication of a shift from the inflammatory phenotype towards the anti-inflammatory macrophage M2 subtype, which also promotes hepatic fibrosis regression [44, 45].

Other notable mRNA expression results include the trend towards increased mRNA expression of molecules involved in lipid handling and storage (SNARE Complex—PLIN1, SNAP23, SYNT5, VAMP4) in the 1144 treatment groups. The results obtained in the mRNA expression of LPL, a major enzyme directly correlated with insulin resistance [46], PPARα, a pro-ketogenic protein involved in insulin-stimulated glucose uptake and fatty acid catabolism [47, 48], and PPARγ, a white adipose tissue marker involved in adipogenic mechanisms and storage of lipids in the liver [49], are also an indication of the 1144-induced improvement. Indeed, PPARs are currently therapeutic targets for NASH, though further research on combination of tissue-specific agonists/antagonists is needed to envisage the use of PPAR-targeted drugs for human metabolic disorders [50].

Noteworthy, the general improvement in liver histological and mRNA expression of markers related to the inflammation, steatosis and fibrosis was substantiated by the significant reduction of liver triglycerides content. In a recently performed 16-week clinical trial with LPCN 1144 in hypogonadal patients with NAFLD, LPCN 1144 reduced liver fat contents (measured by Magnetic Resonance Imaging Proton Density Fat Fraction, MRI-PDFF) by about 40% from baseline and resolved NAFLD in about half of the population [51]. The observation of the reduction of liver triglyceride content in this pre-clinical study supports the findings in the clinical study. A trend of amelioration was also observed for circulating triglycerides and ALP, whereas no effect was found on cholesterol and plasma transferases levels. With regard to bilirubin levels, studies performed in animal models clearly report an increase in bilirubin levels with high fat diet [52, 53]. The increase in bilirubin might be due to obstruction of biliary ducts, and, although the issue is still controversial, it has been reported as an indicator for liver disease [54; https://www.mayoclinic.org]. However, improvements in levels of bilirubin and IGF-1 (both circulating and liver-specific) were observed with 1144 treatments.

Finally, the pattern of IGF-1 mRNA expression between groups was consistent with ones observed in circulating and liver homogenates protein levels. This is in line with the reported literature showing inverse correlation between IGF-1 and fibrosis [55].

Another important observation is that 1144 and 1144 + ALPHA treatments are inducing a drastic reduction of visceral adiposity. Obesity is one of the major comorbidities of hypogonadal males, and several studies have reported that free testosterone levels are low in obese men and inversely correlated with the degree of obesity [56, 57]. The observed excess of visceral fat in HFD and HFD + Veh groups, a major risk factor for the development of MetS and further NASH, is drastically reduced in 1144 and 1144 + ALPHA treatments. The evident reduction of visceral fat observed in 1144 and 1144 + ALPHA groups further highlights the role of visceral fat as a crucial target organ for the compound(s), and suggests a potential clinical benefit of oral testosterone to reduce visceral adiposity.

In comparison with RD, the weight of androgen-target tissues, such as prostate and seminal vesicles, was significantly lower in HFD animals, showing that HFD-related testosterone deficiency reflects not only a biochemical, but also a biological condition of hypogonadism. 1144 treatments not only restored plasma T levels, but also prevented HFD-induced prostate and seminal vesicle atrophy. This normalization by the treatments outlines the importance of prostate and seminal vesicle as physiological targets for testosterone, with circulating total testosterone data also indicating the good absorption of the compound(s).

The reason behind the apparent lack of synergistic effect of LPCN 1144 and α-Tocopherol is not fully elucidated, although it could be speculatively ascribed to the observed lower levels of serum testosterone in rabbits given the combination treatment, compared to 1144 alone, and species differences. Further studies would be warranted to help clarifying this observation.

HFD animals showed a nonsignificant total body weight reduction compared to controls. One possible explanation might concern HFD-driven skeletal muscle hypotrophy, as previously suggested [58, 59]. Our previous studies reported that HFD rabbits showed lower physical endurance, when compared to RD [25], accompanied by HFD-induced skeletal muscle alterations [28]. Likewise, as previously reported with injectable testosterone [27], we observed nonsignificant decreases in body weight of HFD animals treated with 1144. This does not seem an anorexigenic effect, since food intake does not appear to change significantly, and perhaps it can be speculated that testosterone-treated animals are more prone to a higher activity in general. However, a direct comparison of the effect of this new oral formulation of testosterone (LPCN 1144) with injectable testosterone is another major limitation of the present study. It should also be pointed out that estradiol circulating levels were not analyzed during this study, since previous studies from our group demonstrated that HFD induced a two-fold increment in estradiol levels, compared to RD (*p* < 0.001) [27, 60]. Conversely, estradiol levels were fully normalized following testosterone treatment, with a negative and positive correlation, respectively, of testosterone with estradiol levels and number of MetS components, thus also reflecting a negligible aromatase activity in rabbits [27].

In conclusion, the results obtained from this study clearly show, and further confirm, that the 12-week HFD protocol is a validated model for NASH with fibrosis, with all HFD animals displaying established biochemical alterations and liver inflammation, steatosis, ballooning and fibrotic patterns, as well as an increase in the mRNA expression of several inflammatory/fibrotic markers.

The preclinical findings of this study support a therapeutic potential of LPCN 1144 in the treatment of NASH and of hepatic fibrosis. Noteworthy, it has to be recognized that we did not test the effects of this oral androgen in other organs, such as prostate, adipose tissue, skeletal muscle and bladder, which have been demonstrated as important targets of injectable testosterone treatment in the same animal model [23, 27, 28, 33].

Albeit in a preventive experimental model, treatment with oral LPCN 1144, with or without α-Tocopherol, showed a reduction in most of the HFD-induced NASH features, including fibrosis, leading to a significant amelioration at biochemical, molecular and histochemical levels.
